# Predictive Value of Serum 25-Hydroxyvitamin D Levels in the Onset and Glycemic Control of Gestational Diabetes Mellitus

**DOI:** 10.33549/physiolres.935661

**Published:** 2025-12-01

**Authors:** Ling-Ling ZOU, Wu DAI, Jun YE, Yong-Hong CAO, Fa-Hui LV

**Affiliations:** 1 Department of Endocrinology, The Second People’s Hospital of Hefei, Hefei, China; 2 Department of Obstetrics and Gynecology, The Second People’s Hospital of Hefei, Hefei, China

**Keywords:** Blood glucose, Correlation, Gestational diabetes mellitus, Pregnant women, 25-hydroxyvitamin D

## Abstract

This study aimed to evaluate the predictive value of serum 25﷓hydroxyvitamin D (25(OH) D) levels in relation to the onset and glycemic control of gestational diabetes mellitus (GDM). This retrospective study analyzed clinical data of pregnant women who received routine prenatal care and were hospitalized at the Second People’s Hospital of Hefei between January 2023 and January 2025. The study included 200 pregnant women diagnosed with GDM (study group) and 200 gestational age-matched pregnant women with normoglycemia (control group), selected through random sampling. Within the study group, 146 participants exhibited standard glycemic control (Y1 group), while 54 participants exhibited non-standard glycemic control (Y2 group) during hospitalization in the third trimester. Significant differences in serum 25(OH)D levels were observed between the control and study groups across all trimesters (53.82 ± 9.43), (56.73 ± 11.28), (49.65 ± 10.65) nmol/L, and (45.87 ± 8.45), (44.42 ± 10.04), (46.63 ± 9.87) nmol/L (*p* < 0.05). In the second trimester, serum 25(OH)D levels were negatively correlated with the oral glucose tolerance test (OGTT) values in the study group (*p* < 0.05). Comparison of the 25(OH)D levels in the third trimester between the Y1 group (48.95 ± 9.46) and the Y2 group (42.75 ± 10.23) nmol/L indicated that there was no significant statistical difference between the study group and the control group (49.65 ± 10.65 nmol/L) (*p* > 0.05). A receiver operating characteristic curve for first trimester 25(OH)D levels of pregnant women in the study group yielded an area under the curve of 0.84. Lower serum 25(OH)D levels were associated with an elevated risk of developing GDM and with poorer glycemic control in affected women. These findings indicate that first trimester serum 25(OH)D levels may serve as a valuable biomarker for the early prediction and management of GDM.

## Introduction

Gestational diabetes mellitus (GDM) is a pregnancy-related metabolic disorder characterized by glucose intolerance first identified during gestation, following the exclusion of pre-existing diabetes mellitus. While abnormalities in carbohydrate metabolism are central to the pathophysiology of GDM, other metabolic alterations may also contribute, although the underlying mechanisms remain incompletely understood. Currently, the prevalence of GDM in China is estimated to range from 10 % to 15 %, placing it in the mid-to-high range globally [1]. Pregnant women diagnosed with GDM are at an increased risk of adverse maternal and perinatal outcomes, including fetal macrosomia, spontaneous abortion, preterm birth, and neonatal metabolic complications. Additionally, epidemiological data indicate a higher incidence of obesity and cardiovascular disorders in offspring born to women with GDM compared to those born to women without glucose abnormalities during pregnancy [2]. Given these risks, identifying modifiable risk factors and predictive markers for GDM remains a priority in perinatal research. Vitamin D, a critical nutrient in fetal skeletal development, has garnered increasing attention for its potential association with GDM. Studies conducted in various regions, including Taiwan, have explored the relationship between serum vitamin D levels and GDM onset [3]. Among vitamin D metabolites, 25﷓hydroxyvitamin D (25-(OH)D) is considered the most clinically relevant biomarker due to its stability, extended half-life, and accessibility for routine measurement [4]. As fetal vitamin D status is entirely dependent on maternal supply, and 25(OH)D readily crosses the placenta, its role in fetal development is of particular relevance. However, existing studies have yielded inconsistent findings regarding the association between serum 25(OH)D concentrations and the risk of GDM [5].

Given the variability in existing evidence, this study aimed to evaluate the predictive value of serum 25(OH)D levels for the development and glycemic control of GDM through a retrospective analysis of clinical data from pregnant women receiving routine prenatal care at a single institution. The objective was to further clarify the nature of this relationship and to provide a theoretical foundation for early identification and intervention strategies aimed at reducing the incidence of GDM.

## Methods

### General data

A retrospective analysis was conducted on clinical records of pregnant women who received routine prenatal care and were hospitalized at the Second People’s Hospital of Hefei between January 2023 and January 2025. A total of 400 participants were included in the analysis: 200 with normoglycemic pregnancies (control group) and 200 diagnosed with GDM (study group). Within the study group, 146 participants achieved standard glycemic control (Y1 group), while 54 exhibited non-standard glycemic control (Y2 group) during hospitalization in the third trimester.

Inclusion criteria: Participants were eligible for inclusion if they met all of the following criteria: (1) Initiation of prenatal care before 13 weeks of gestation. (2) Confirmation of a singleton pregnancy (3) Completion of routine prenatal evaluations throughout gestation. (4) Completion of an oral glucose tolerance test (OGTT) between 24 and 28 weeks of gestation. (5) Delivery at the study hospital during the third trimester.

Exclusion criteria: Participants were excluded if they had any of the following: A documented history of pre-existing chronic conditions such as type 1 or type 2 diabetes mellitus, chronic hypertension, or thyroid dysfunction, diagnosis of medical complications during pregnancy such as gestational hypertension, chronic use of vitamin D supplements, or the presence of other chronic systemic disorders.

### Research methods

Serum 25(OH) D concentrations were measured at three gestational time points: first trimester (11–13 + 6 weeks), second trimester (24–28 + 6 weeks), and third trimester (≥ 37 weeks). Fasting peripheral blood samples were collected for analysis. Quantification of serum 25(OH) D was performed using the Roche e602 electrochemiluminescence immunoassay system.

OGTT was conducted in accordance with the diagnostic criteria outlined in the 10th edition of Obstetrics and Gynecology (People’s Medical Publishing House). Fasting plasma glucose and plasma glucose levels at 1 hour and 2 hours post-glucose ingestion were recorded. The diagnosis of GDM was based on these standardized guidelines.

### Statistical methods

All statistical analyses was conducted using SPSS software, version 24.0. Data with a normal distribution were expressed as mean ± standard de*via*tion (χ̄ ± s). Comparisons between two independent groups were conducted using the independent-samples *t*-test while comparisons across multiple groups were analyzed using Analysis of Variance (ANOVA). Categorical variables were compared using the chi-squared (χ^2^) test.

The Pearson’s correlation coefficient was used to assess the relationship between serum 25(OH) D levels and venous blood glucose concentrations at various OGTT time points. The predictive utility of 25(OH) D for GDM was evaluated using receiver operating characteristic (ROC) curve analysis. A *p*-value of < 0.05 was considered indicative of statistical significance.

## Results

### General data

No statistically significant differences were observed between the study group (study group) and the control group with respect to age, pre-pregnancy body mass index (BMI), gestational age at the time of blood sampling, gravidity, or parity (Table 1). These findings indicate that the two groups were comparable with respect to baseline demographic and clinical characteristics, thereby minimizing the influence of potential confounding factors such as pre-pregnancy BMI, on serum 25(OH) D concentrations.

### Comparison of serum 25(OH) D levels across Trimesters

Statistically significant differences in mean serum 25(OH) D concentrations (nmol/L) were observed between the control and study groups across all three trimesters (*p* < 0.05) (Table 2). In all three gestational periods, participants in the study group exhibited consistently lower 25(OH)D levels compared to those in the control group, indicating a potential association between decreased vitamin D status and the presence of GDM.

### Correlation between serum 25(OH)D and glycemic parameters in the second trimester

Pearson’s correlation analysis demonstrated a statistically significant inverse correlation between serum 25(OH)D concentrations and fasting, 1-hour postprandial, and 2-hour postprandial plasma glucose levels during the second trimester in the GDM group (*p* < 0.05) (Table 3).

These results indicate that lower serum 25(OH)D concentrations are associated with higher glucose levels, supporting a negative correlation between vitamin D status and glycemic control.

### Association between glycemic control and serum 25(OH)D

In the third trimester, serum 25(OH)D concentrations were significantly lower in the non-standard glycemic control group (Y2) compared to the standard glycemic control group (Y1) (*p* < 0.05) (Table 4). However, no statistically significant differences were identified between both study subgroups and the control group (*p* > 0.05). These findings indicate that adequate glycemic control may be associated with more favorable serum 25(OH)D levels in women with GDM in late pregnancy.

### Predictive value of first-trimester 25(OH)D for GDM

**Fig. 1:**
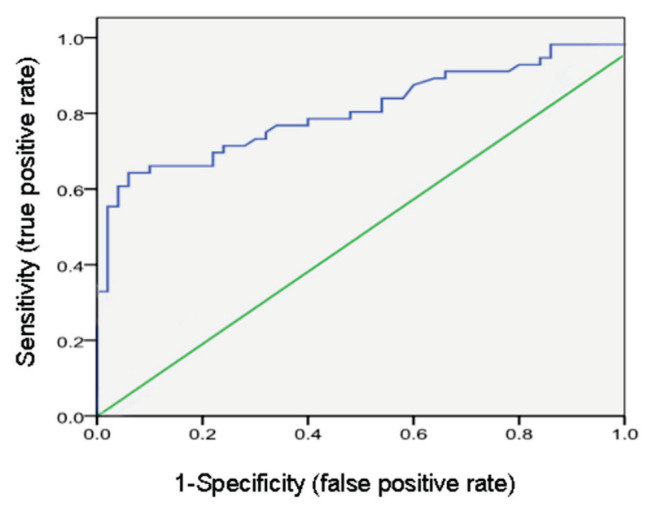
Predictive value of first-trimester serum 25(OH)D for the onset of GDM

ROC curve analysis for first-trimester serum 25(OH)D concentrations in the study group yielded an area under the curve (AUC) of 0.82, indicating good discriminatory ability (Fig. 1). The model demonstrated a sensitivity of 75.3 % and a specificity of 62.4 %, supporting the potential utility of first-trimester 25(OH)D as an predictive biomarker for the development of GDM.

## Discussion

GDM is a common metabolic complication of pregnancy associated with an increased risk of adverse maternal and perinatal outcomes. Although the precise pathophysiological mechanisms underlying GDM remain under investigation, growing evidence supports its link to insulin resistance and β-cell dysfunction. The rising prevalence of GDM in recent years may be attributed to improvements in living standards, advancements in prenatal care, increased awareness of GDM, and standardized screening protocols. In response to this trend, there has been a growing body of research aimed at identifying modifiable risk factors for GDM, with the goal of mitigating associated maternal and fetal complications. Vitamin D deficiency has emerged as a candidate factor due to its recognized role in insulin secretion and insulin signaling pathways. A significant number of individuals with various forms of diabetes exhibit reduced vitamin D levels, suggesting a possible contribution to dysregulated glucose metabolism [6].

Among vitamin D metabolites, 225(OH)D is considered the most reliable biomarker for assessing vitamin D status, due to its stable serum concentration, prolonged half-life and practicality in clinical measurement. Previous studies have reported associations between reduced 25(OH)D concentrations and elevated blood glucose levels, insulin resistance, and increased GDM risk [7]. In the present study, a statistically significant difference in 25(OH)D deficiency was observed in the first trimester among women later diagnosed with GDM during the second trimester, as determined by OGTT. These findings suggest that adequate supplementation of 25(OH) D during early pregnancy may contribute to a reduced incidence of GDM. Moreover, serum 25(OH)D levels demonstrate potential sensitivity in predicting the onset of glucose metabolism disorders, consistent with previous findings [8–10].

In this study, the relationship between serum 25(OH)D levels and GDM across different gestational periods was assessed, elucidating the correlation between circulating glucose levels and 25(OH)D concentrations. The findings support the recommendation of vitamin D supplementation during pregnancy and emphasize the importance of maintaining sufficient serum 25(OH) D levels to reduce the likelihood of GDM. Routine measurement of serum 25(OH) D is therefore recommended as part of standard prenatal assessments.Vitamin D synthesis in humans is predominantly influenced by cutaneous exposure to sunlight, physical activity, and dietary intake. Societal shifts and technological changes have contributed to lifestyle patterns characterized by prolonged indoor activity and limited exposure to natural sunlight, thereby reducing the skin’s capacity to synthesize vitamin D. However, increased exposure to sunlight remains one of the most effective and accessible strategies for enhancing endogenous vitamin D production and preventing deficiency [11]. It is estimated that approximately 90 % of the vitamin D present in the human body is synthesized through sunlight-induced conversion in the skin.

In addition to limited sunlight exposure, contemporary dietary patterns often lack sufficient vitamin D content. This deficiency is particularly pronounced during the first trimester, when pregnant women often experience pregnancy-related nausea and vomiting, leading to reduced dietary intake, a limited variety of foods, and suboptimal nutritional adequacy. In the second trimester, physiological changes such as delayed gastrointestinal motility may lead to a preference for low-fat, easily digestible foods such as starches and grains, which are generally poor sources of vitamin D. These dietary patterns further contribute to the risk of vitamin D deficiency during pregnancy.

Findings from this study indicate that vitamin D supplementation during early pregnancy may play an important role in reducing the incidence of GDM. Therefore, in addition to routine folic acid supplementation in early pregnancy, the inclusion of vitamin D supplementation warrants increased clinical attention. Adjustments to dietary patterns, increased exposure to natural light, and appropriate vitamin D supplementation are all critical in reducing the incidence of pregnancy-related complications.

Physiological demands for vitamin D and calcium increase substantially during pregnancy to support fetal skeletal development, maternal bone mineralization, and calcium homeostasis [12]. If unaddressed, prolonged vitamin D deficiency may adversely affect fetal skeletal and dental development and could potentially impact bone health later in life. Consequently, exogenous supplementation of vitamin D and calcium is essential to support optimal maternal and fetal outcomes [13].

Furthermore, reduced maternal estrogen levels have been hypothesized to contribute to both vitamin D deficiency and the development of GDM, given the role of estrogen in modulating bone metabolism signaling pathways. Estrogen is necessary for maintaining bone mass and supporting skeletal development. Recent studies have also identified a potential association between vitamin D deficiency and hypertensive disorders of pregnancy, further underscoring a broader role for vitamin D in maternal health [14].

In the present study, serum 25(OH)D concentrations were significantly lower in pregnant women with GDM compared to those with normoglycemic pregnancies. Moreover, first-trimester serum 25(OH)D levels demonstrated moderate predictive value for the subsequent development of GDM. This finding aligns with previous research by Ziling et al., who reported that second-trimester 25(OH)D levels also held predictive value for GDM onset [6]. However, findings by Changhong et al. diverge from these results, indicating that glycated hemoglobin (HbA1c) in early pregnancy may be a reliable predictor [15]. Specifically, their analysis indicated that the AUC for first trimester 25(OH)D was below 0.5, suggesting limited predictive utility when compared with HbA1c. These discrepancies underscore the need for further investigation and validation through studies involving larger, more diverse populations.

An inverse correlation has been observed between serum 25(OH)D_3_ levels and fasting plasma glucose, indicating a potential role for 25(OH)D_3_ in modulating glucose metabolism under fasting conditions. In this study, serum 25(OH)D concentrations measured at the time of GDM diagnosis *via* OGTT were significantly associated with the presence of GDM. Additionally, negative correlations were identified between serum 25(OH)D levels and multiple glycemic indices. Within the GDM cohort, pregnant women who achieved glycemic control during the third trimester exhibited higher serum 25(OH)D concentrations than those with suboptimal control. These findings indicate that correction of vitamin D deficiency may contribute to improved glycemic control during pregnancy.

This evidence supports the theoretical basis for calcium and vitamin D supplementation during the first and second trimesters to reduce GDM risk and improve metabolic outcomes. However, as this study was conducted at a single institution, the generalizability of the findings is limited. Further validation through large-sample, multicenter trials is warranted to strengthen external validity and account for potential confounding variables.

## Conclusion

Overall, serum 25(OH)D demonstrates considerable potential as a predictive biomarker for GDM. Its integration into routine prenatal screening protocols may facilitate earlier identification of pregnant women at elevated risk, allowing for timely initiation of vitamin D supplementation and appropriate clinical management. Periodic assessment of serum 25(OH)D concentrations throughout pregnancy may contribute to the reduction of pregnancy-related complications, mitigating adverse maternal-fetal outcomes, and promote optimal perinatal health.

## Figures and Tables

**Table 1:** Baseline characteristics of the study and control groups

Groups	Age (years)	Number of pregnancies	Number of deliveries	Pre-pregnancy body mass index	Gestational age of blood collection		

					First trimester	Second trimester	Third trimester
*Control group (n = 200)*	28.45±5.63	1.59±0.25	1.31±0.32	22.03±1.43	12.45±1.36	24.12±1.63	38.23±1.06
*Study group (n = 200)*	28.56±5.47	1.62±0.21	1.28±0.35	21.65±1.38	12.54±1.39	24.33±1.58	38.34±1.12
*t value*	1.32	1.45	2.34	1.23	1.43	1.58	1.27
*p value*	0.25	0.21	0.11	0.28	0.25	0.36	0.52

**Table 2:** Comparison of 25(OH)D concentrations and glycemic indices across trimesters

Groups	25(OH)D (nmol/L)			OGTT (mmol/L)		

	First trimester	Second trimester	Third trimester	FPG	1hPBG	2hPBG
*Control group (n = 200)*	53.82±9.43	56.73±11.28	49.65±10.65	4.35±0.41	7.46±1.74	6.35±0.73
*Study group (n = 200)*	45.87±8.45	44.42±10.04	46.63±9.87	5.12±0.48	10.02±1.64	8.67±1.35
*t value*	31.014	33.234	34.473	25.543	26.435	31.745
*p value*	0.001	0.001	0.001	0.001	0.001	0.001

**Table 3:** Correlation between serum 25(OH)D levels and glycemic parameters in the second trimester (GDM group)

Items	OGTT (mmol/L)		

	FPG	1hPBG	2hPBG
*r value*	−0.067	−0.098	−0.075
*p value*	0.021	0.013	0.001

**Table 4:** Comparison of 25(OH)D concentrations and glycemic control in GDM subgroups and control group

Groups	Cases (n)	25(OH)D (nmol/L)	FPG (nmol/L)	2hPBG (nmol/L)
*Study group Y1 group*	146	48.95±9.46	4.45±0.49	5.93±0.67
*Study group Y2 group*	54	42.75±10.23	5.42±0.53	6.47±1.25
*Control group*	200	49.65±10.65	4.75±0.38	5.89±0.51
*F value*		4.352	3.562	3.241
*p value*		0.001	0.001	0.001

## Abbreviations

GDM: Gestational diabetes mellitus
25-(OH)D: 25 hydroxyvitamin D
OGTT: Oral glucose tolerance test
ROC: Receiver Operating Characteristic
HbA1c: Glycated Hemoglobin
AUC: Area Under the Curve
